# Investigating Immune Checkpoint Inhibitor-Induced Pancreatic Injury: When to Discontinue Cancer Therapy

**DOI:** 10.3390/metabo15060385

**Published:** 2025-06-10

**Authors:** Enrico Celestino Nista, Sara Sofia De Lucia, Sebastiano Archilei, Jacopo Iaccarino, Giulia Piccirilli, Alberto Nicoletti, Angela Saviano, Antonio Gasbarrini, Veronica Ojetti

**Affiliations:** 1 Department of Medical and Surgical Sciences, Università Cattolica Sacro Cuore, Fondazione Policlinico Universitario A. Gemelli IRCCS, 00168 Rome, Italy; enricocelestino.nista@policlinicogemelli.it (E.C.N.); sarasofia.delucia@guest.policlinicogemelli.it (S.S.D.L.); sebastiano.archilei01@icatt.it (S.A.); jacopo.iaccarino01@icatt.it (J.I.); giulia.piccirilli01@icatt.it (G.P.); alberto.nicoletti@policlinicogemelli.it (A.N.); antonio.gasbarrini@policlinicogemelli.it (A.G.); 2 Department of Emergency, Anesthesiological, and Reanimation Sciences, Università Cattolica Sacro Cuore, Fondazione Policlinico Universitario A. Gemelli IRCCS, 00168 Rome, Italy; angela.saviano@policlinicogemelli.it; 3 San Carlo di Nancy GVM Care and Research, Department of Internal Medicine, UniCamillus International University, 00131 Rome, Italy

**Keywords:** immune checkpoint inhibitors, immune-related adverse events, metabolic perturbations, pancreatic injury

## Abstract

**Background/Objectives:** The increasing use of immune checkpoint inhibitors (ICIs) in cancer treatment has led to a rise in immune-related adverse events (irAEs), including pancreatic injury. While current guidelines suggest that baseline monitoring of amylase and lipase levels is not necessary, it remains common in clinical settings, leading to confusion regarding their interpretation and management. This practice may lead to confusion, especially when patients exhibit isolated mild elevations of amylase and lipase, which may not always correlate with clinical pancreatitis. In contrast, significant elevations in these enzymes warrant further investigation, including imaging to assess the presence of pancreatitis. **Methods:** This review aims to provide a clearer framework for clinicians in managing ICI-induced pancreatic injury, promoting consistency in practice, and improving patient outcomes by reducing unnecessary interruptions to ICI therapy. **Results:** It is critical to distinguish between the severity of pancreatitis to guide management. **Conclusions:** Considering the expected rise in the use of immune ICIs, it is crucial to increase awareness about the potential for pancreatic injury associated with these treatments.

## 1. Immune Checkpoint Inhibitor-Induced Pancreatic Injury

### Introduction, Epidemiology, and Risk Factors

The human immune system is capable of detecting threats such as signs of malignancy. A revolutionary approach to take advantage of this power has been found in modulating immune regulatory mechanisms to enhance antitumor response [1]. A potential target for drug development has been identified in T cell co-receptors as they can either promote or downregulate T cell activation. Two inhibitory T cell co-receptors, cytotoxic T-lymphocyte antigen 4 (CTLA-4) and programmed cell death 1 (PD-1), along with ligand programmed cell death ligand 1 (PD-L1), act through two independent signaling pathways to downregulate the immune system [2,3,4]. PD-1 is a negative co-stimulatory receptor belonging to the CD28 immunoglobulin superfamily. It functions as a potent inhibitor of effector T cell responses and is primarily expressed on CD8^+^ T cells, though it is also found on B cells, NK cells, T regulatory cells (Tregs), and bone marrow–derived suppressor cells. PD-1 has two known ligands, PD-L1 (B7-H1/CD274) and programmed cell death ligand 2 (PD-L2) (B7-DC/CD273). When PD-1 binds to these ligands, it transmits an inhibitory signal through the T cell receptor, suppressing T cell activation and cytokine production. This mechanism contributes to tumor immune evasion by dampening antitumor immune responses. Elevated PD-1 expression also influences the proliferation and differentiation of Tregs, further promoting peripheral immune tolerance and weakening the immune system’s ability to target cancer cells [5]. Cytotoxic T-lymphocyte antigen 4 (CTLA-4/CD152) and CD28 are homologous receptors expressed on CD4^+^ and CD8^+^ T cells, but they play opposing roles in T cell activation. Both bind to the same ligands—CD80 and CD86—on antigen-presenting cells (APCs). CD28 provides essential co-stimulatory signals in conjunction with T cell receptor (TCR) activation, promoting T cell proliferation and cytokine production. In contrast, CTLA-4 binds these ligands with higher affinity, delivering inhibitory signals that suppress T cell responses and help maintain immune tolerance. Furthermore, CTLA-4 internalizes CD80 and CD86 via trans-endocytosis, reducing their availability for CD28 binding and further dampening immune activation. This regulatory mechanism explains why a stimulatory and inhibitory receptor share the same ligands and underscores how the CD28/CTLA-4 axis acts as an immune rheostat, modulating T cell responses based on the immunologic context [6]. Immune checkpoint inhibitors (ICIs), such as PD-1/PD-L1 and anti-CTLA-4 therapies, demonstrated clinical efficacy in the treatment of various malignancies including melanoma [7], renal cell carcinoma [8], otorhinolaryngological cancers, and lung cancer as non-small cell lung cancer [9]. These agents work by modulating immune checkpoints, enhancing T cell-mediated antitumor effects by targeting CTLA-4, PD1, and PD-L1 [10]. Anti-CTLA-4 antibodies, such as Ipilimumab, block CTLA-4 function, restoring co-stimulation through CD28 and enhancing T cell-mediated antitumor immunity. In the tumor microenvironment, cancer cells often upregulate PD-L1 to engage PD-1 on effector T cells, leading to T cell exhaustion and impaired antitumor responses. This interaction is a critical mechanism of immune escape exploited by many malignancies. ICIs targeting the PD-1/PD-L1 axis, such as Nivolumab, Pembrolizumab, and Atezolizumab, restore T cell function by blocking this inhibitory pathway. While highly effective, ICIs are associated with distinct adverse inflammatory reactions known as immune-related adverse events (irAEs) [11,12], which can range from mild self-limiting symptoms to severe life-threatening events. The etiology of irAEs is not fully understood but has been hypotesized as the result of an overactivation of the immune system and a lack of proper immune regulation, the same process that makes ICIs effective against cancer [1,12]. Immune-related adverse events (IrAEs) can impact nearly any organ system, with the skin, gastrointestinal tract, lungs, liver, and endocrine system being the most commonly affected [13]. Among them, pancreatic irAEs, though less common than the involvement of other organs, represent a clinically significant concern because of their potential impact on both patient quality of life and cancer treatment continuity.

ICI-induced pancreatic injury (ICI-PI) represents a rare complication that occurs in up to 4% of patients [14], whose clinical presentation could be quite heterogeneous, from an asymptomatic increase in serum lipase and amylase increase to acute pancreatitis [13,15]. The development of pancreatitis occurred anywhere from 20 days to over a year following the initiation of ICI therapy, depending on the individual case. Common Terminology Criteria for Adverse Events (CTCAE) version 4.0 was used to describe ICI-PI, which can be classified into grades 1–4 according to the serum pancreatic enzyme increase [16] as follows: grade 1 upper limit of normal (ULN) × 1.5 ULN; grade 2 > 1.5–2.0 × ULN; grade 3 > 2.0–5.0 × ULN; grade 4 > 5.0 × ULN. Grade 5 is classified as mortality related to pancreatitis [17]. The latest version of CTCAE (version 5.0) defines ICI-PI based on the degree of serum enzyme elevation and symptoms, e.g., grade 1 and grade 2 result in asymptomatic pancreatitis with an enzyme elevation or radiologic findings only, grade 3 requires the presence of severe pain, vomiting, and the need for medical intervention (analgesia, nutritional support); grade 4 is a life-threatening condition with urgent intervention indicated, and grade 5 corresponds to death [18]. In their meta-analysis, Zhang et al. [19] investigated the incidence of ICI-PI, reporting an overall pancreatic injury incidence of 2.22% across all grades, which was lower than in previous similar studies [16,20,21]. Furthermore, this study aligns with previous ones, confirming that the incidence of pancreatic injury with combined regimens involving both anti-PD-1/PD-L1 and anti-CTLA-4 agents is significantly higher compared with monoimmunotherapy [16,20,22]. Yasuki et al. confirmed that both simultaneous and dual immunotherapy were associated with a higher overall rate of pancreatic injury than monoimmunotherapy [17]. Although the overall incidence of total pancreatic injury did not significantly differ between simultaneous and asynchronous immunotherapy, severe cases (grade ≥ 3) were more frequently observed in the simultaneous immunotherapy group [17]. The relatively high rates of simultaneous and dual immunotherapy in this large cohort, based on real-world clinical practice, may explain the higher incidence of ICI-PI observed [17]. An analysis of various types of immune checkpoint inhibitors showed that PD-L1 inhibitors were associated with a higher incidence of PI (3.01%) compared with CTLA-4 inhibitors (2.92%) and PD-1 inhibitors (2%), challenging the earlier belief that CTLA-4 inhibitors were the primary contributors of ICI-PI [16,20,23,24,25]. According to the study, the highest incidence of ICI-PI was observed with Pembrolizumab, (7.23%), consistent with findings from a previous study [26]. Moreover, several studies have explored the risk factors associated with ICI-PI [13,16,19,20]. George et al. [16] highlighted that CTLA-4 inhibitors, combination therapy, and malignant melanoma can be considered high-risk factors for ICI-PI [26]. Nagao et al. investigated in a large-scale multicenter study the incidence, risk factors, and clinical course of ICI-PI in Japan. During risk factors analysis, they included several parameters not considered in previous studies, revealing that renal cancer, melanoma, CTLA-4 inhibitors, multiple ICI monotherapies, combination ICI therapy, and irAEs in other organs—particularly the liver and endocrine system—were significantly associated with a higher risk of ICI-PI. In addition, the history of IFN therapy was associated with the activation of T cell-based immunity, which may promote autoimmune-like reactions, thus serving as another risk factor for ICI-PI [27]. Table 1 summarizes the incidence rates of immune-related pancreatitis and pancreatic injury, stratified by monotherapy versus combination therapy and by inhibitor class. Overall, ICI-PI is a rare occurrence, with presentations ranging from asymptomatic cases to acute pancreatitis, potentially leading to the development of a pseudocyst and subsequent endocrine and exocrine insufficiency [13]. Prompt identification and management of pancreatic involvement from ICIs are essential to improve patient outcomes and balance the treatment of irAEs with the continuation of life-saving immunotherapy.

## 2. Pathogenesis of ICI-Induced Pancreatic Injury

**Figure 1:**
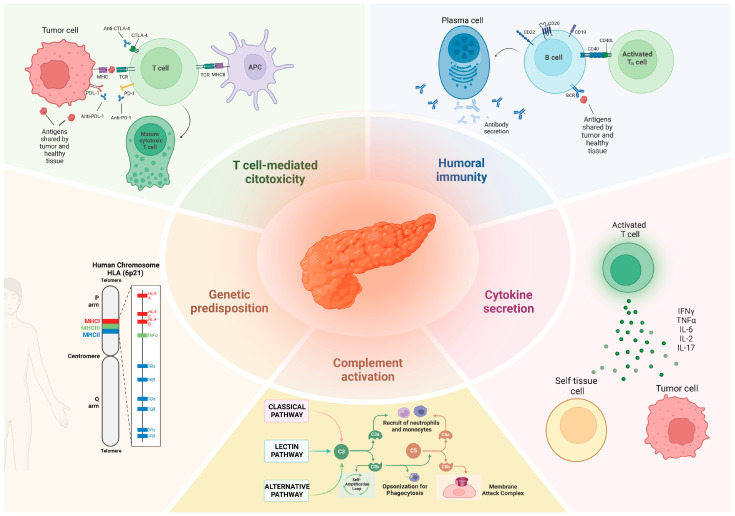
Pathogenesis of immune-related adverse events: T cell-mediated cytotoxicity involves the activation of CD8^+^ T cells via the presentation of antigens shared by the tumor and by self-tissues to the T cell receptor (TCR), induced by tumor cells directly or mediated by antigen-presenting cells (APC) through major histocompatibility complex (MHC). Humoral immunity consists of the transformation of B cells into plasma cells capable of producing autoantibodies. A third mechanism involves the secretion of cytokines and chemokines by activated T cells. Complement cascade activation has been described as a possible mechanism in some cases of hypophysitis induced by anti-CTLA-4 therapy. Finally, a fifth mechanism involves genetic predisposition in individuals carrying specific human leukocyte antigen (HLA) risk alleles.

Pancreatitis is an inflammatory condition of the pancreas that can manifest as either acute or chronic. Its pathogenesis is primarily driven by the premature activation of pancreatic enzymes, particularly trypsinogen, leading to pancreatic tissue injury and subsequent autodigestion of the gland. This damage triggers the release of damage-associated molecular patterns (DAMPs), fostering a pro-inflammatory microenvironment, promoting necroptosis, and increasing intestinal permeability. Consequently, the translocation of gut bacteria into the bloodstream further amplifies the inflammatory response, contributing to disease progression [28]. ICI-mediated toxicity, and immune-related adverse events (irAEs), share pathogenetic mechanisms with autoinflammatory and autoimmune diseases [29]. Given the limited epidemiology of ICI-associated pancreatitis (ICI-AP), and the relatively recent use of ICIs in oncology, the specific pathogenetic mechanism underlying ICI-induced pancreatic injury still needs to be cleared. However, five distinct pathways have been identified through which ICIs can induce irAEs (Figure 1). The first mechanism involves cellular autoimmunity, characterized by heightened T cell activation, which enhances cytotoxic activity against antigens common to both tumor cells and self-tissues [29]. The second mechanism involves humoral immunity, where B cell activity plays a pivotal role not only in antitumor responses but also in the unintended production of auto-antibodies [30]. This process contributes to the development of irAEs, which can affect either single or multiple organs, depending on the tissue specificity of the auto-antibodies. The third mechanism involves the increased secretion of cytokines and chemokines driven by T cell activation [31]. Specific cytokines appear to be associated with distinct irAEs; for instance, IL-17 has been linked to ICI-induced colitis, IL-1 and IL-6 to skin lesions, and IL-1β and IL-2 to thyroiditis [31]. The fourth mechanism involves complement activation, which has been specifically described in cases of hypophysitis induced by anti-CTLA-4 therapy. This occurs when anti-CTLA-4 antibodies bind to CTLA-4 in healthy tissues, initiating the complement cascade [32]. The fifth mechanism involves genetic predisposition. Studies have shown that ICI-induced arthritis and diabetes occur predominantly in individuals carrying HLA-DR risk alleles, suggesting a genetic susceptibility to these irAEs [33,34]. The exact mechanism underlying pancreatic injury caused by immune checkpoint inhibitors remains largely unclear. However, pancreatic biopsies in affected patients have shown infiltration of CD3+ T cells, particularly CD8^+^ T cells. This infiltration may result from the blockade of inhibitory T cell signals by ICIs, leading to damage in both endocrine and exocrine pancreatic tissue [35]. Furthermore, lymphoid infiltration characterized by TIA1+ and granzyme B+ cells have been observed in affected tissue, as shown by immunohistochemical analysis [36]. In a recent paper, Chen et al. described common histopathological findings in tissues from patients with ICI-associated pancreatitis (ICI-AP) including acinar-centric mixed inflammatory infiltrates, atrophy, and fibrosis; additionally, features such as storiform fibrosis, edema, and acinar-to-ductal metaplasia were not as frequently observed [37]. These interesting hallmarks could also be useful to differentiate between ICI-AP and AIP, which is more often characterized by IgG4 plasma cells.

## 3. Clinical Features of ICI-Induced Pancreatic Injury

Clinically, ICI-PI can be either symptomatic or asymptomatic. When symptomatic, ICI-PI often presents similarly to acute pancreatitis, with abdominal pain being the most common symptom, typically localized in the epigastric region and radiating to the back. Patients may also experience symptoms such as diarrhea, nausea, vomiting, and fever [13,38]. Interestingly, in some cases, ICI-induced pancreatic injury has been described as presenting signs of exocrine insufficiency. Affected patients may experience weight loss despite adequate caloric intake, unformed stools, steatorrhea, and occasional abdominal pain [39]. The pathophysiology of this phenomenon is thought to involve significant inflammation of the pancreatic gland, leading to tissue atrophy. Activated and increased CD8^+^ T cells have been attributed to the immunotherapy-induced infiltration inside and around the pancreas, causing damage to pancreatic cells. This damage decreases the number of pancreatic ductal and acinar cells (exocrine pancreas), resulting in pancreatic atrophy [39]. Although rare, pancreatic exocrine insufficiency secondary to ICIs can be diagnosed when fecal pancreatic elastase levels are found to be low, and symptoms can often be alleviated with oral enzyme supplementation. It is important to note that diarrhea in patients undergoing ICI therapy should be carefully investigated, as it needs to be differentiated from ICI-induced colitis. Early diagnosis is crucial to avoid unnecessary steroid treatment.

Nevertheless, some patients were observed to have pancreatitis highlighted in imaging studies while remaining entirely asymptomatic.

## 4. Diagnostic Workup of ICI-Induced Pancreatic Injury

Mild asymptomatic increases of pancreatic enzyme levels may occur in patients undergoing immunotherapy with immune checkpoint inhibitors (ICIs), without necessarily indicating the development of pancreatitis [26]. Table 2 summarizes clinically significant immune-related pancreatitis from isolated pancreatic enzyme elevations. Given this, it is important to differentiate between checkpoint inhibitor-induced pancreatic injury (ICI-PI) and ICI-associated pancreatitis (ICI-AP). In cases where the patient remains asymptomatic, routine monitoring of pancreatic enzymes is generally not recommended, unless there is a clinical suspicion of ICI-PI [26]. ICI-associated pancreatitis (ICI-AP) is diagnosed when at least two out of the three conventional criteria for acute pancreatitis are met. The first criterion is the presence of clinical features, as previously mentioned. The second criterion is a threefold increase in serum pancreatic enzymes, with lipase being the most accurate marker. The third criterion involves imaging findings, typically observed through computed tomography (CT). Common CT signs of acute pancreatitis include enlargement of the pancreas, indicating edema, segmental hypoenhancement, and stranding in the peripancreatic fat [40]. Das JP et al. reported that CT imaging in cases of pancreatitis most frequently resembled acute pancreatitis (80%), followed by features suggestive of autoimmune pancreatitis (16%), with a smaller subset (4%) exhibiting a mixed pattern of both [40]. Magnetic resonance imaging (MRI) can reveal early signs of pancreatic damage, which may appear as a decrease in signal intensity and restricted diffusion. In some cases, MRI may also show features suggestive of autoimmune pancreatitis (AIP), including enhancement during the late phase of gadolinium contrast and signal restriction on diffusion-weighted images. Additionally, stenosis of the main pancreatic duct at the pancreatic head may be observed [41,42]. Since 18F-fluorodeoxyglucose positron emission tomography (FDG-PET) is frequently used to assess the response to the treatment of primary tumors, cases of ICI-associated pancreatitis (ICI-AP) have been identified based on the presence of significant 18F-FDG uptake in the pancreas [43]. Endoscopic ultrasound (EUS) also plays a crucial role in the diagnosis, revealing diffuse hypoechoic enlargement of the pancreas with patchy and heterogeneous parenchyma. These features are characteristic of both ICI-AP and AIP [44] and there are no established specific ultrasound imaging features for ICI-associated pancreatitis. Differentiating focal-type pancreatitis from pancreatic cancer is particularly challenging since both present as hypoechoic masses. In contrast to pancreatic cancer, autoimmune pancreatitis is often characterized by multiple masses; additionally, the duct penetration sign, which represents the pancreatic duct within the affected parenchyma, represents a feature of autoimmune pancreatitis.

Moreover, EUS with fine-needle aspiration (FNA) in cases of ICI-PI has revealed infiltration of inflammatory cells, predominantly consisting of neutrophils and T cells. Notably, there is a predominance of CD8^+^ T cells over CD4^+^ T cells in the pancreatic tissue [45]. Interestingly, fine-needle biopsy (FNB) via EUS could be useful for ruling out AIP in the absence of IgG4 plasma cells. However, it is important to consider the risks associated with this invasive procedure when deciding whether to proceed with it [46].

Typically, endoscopic retrograde cholangiopancreatography (ERCP) is not utilized for diagnosing acute pancreatitis, and there are limited reports regarding the use of ERCP for ICI-AP.

## 5. Management of ICI-Induced Pancreatic Injury

The infrequent occurrence of ICI-induced pancreatic injury, combined with the lack of robust evidence, has limited the development of a well-defined and effective management strategy. The latest National Comprehensive Cancer Network (NCCN) guidelines represent the only formal recommendations available to guide the treatment of ICI-induced pancreatic injury but are all recommendations of category 2A (NCCN consensus ≥ 85% support of the panel but based upon lower level evidence). According to these, asymptomatic patients with elevated pancreatic enzymes and normal pancreatic imaging should continue receiving ICI therapy without any intervention [47]. On the other hand, patients with manifested ICI-related pancreatitis must be carefully evaluated to determine the necessity of hospitalization. ICI therapy should be discontinued, and intravenous (IV) fluid replacement should be initiated, along with supportive management for pain and nausea. The need for hospitalization and IV fluid replacement is observed in 90% of patients with ICI-associated pancreatitis (ICI-AP), compared with those with asymptomatic pancreatic injury [48,49]. Furthermore, according to NCCN guidelines, patients with pancreatitis should begin treatment with glucocorticosteroids at doses of 0.5–1 mg/kg/day for moderate cases and 1–2 mg/kg/day for severe cases. However, a large retrospective study has shown that, while intravenous hydration reduces the risk of long-term adverse outcomes, the effectiveness of steroids—recommended by NCCN guidelines as the cornerstone therapy for moderate to severe ICI-associated pancreatitis—is not well established. In fact, among 2279 cases, no statistically significant differences were observed in the duration of symptoms or hospitalization for patients with ICI-AP, regardless of whether corticosteroids were used [13,47]. In addition, the response to steroid therapy may be suboptimal, as it does not always effectively reduce pain severity or duration, improve biochemical markers, or prevent pancreatic volume loss [50]. If no satisfactory response is observed within 72 h, the ASCO guidelines, based on limited evidence, observational data, case reports, and clinical experience, suggest increasing the steroid dosage and/or adding immunosuppressive such as infliximab (5 mg/kg) or mycophenolate mofetil (1 g twice daily intravenously) [48]. Other immunosuppressants, including cyclophosphamide or intravenous immunoglobulin (IVIG), may be considered in select cases [48]. Currently, the most promising immunosuppressive agents used as an alternative to steroids are TNF-α antagonists, particularly Infliximab. This agent has already demonstrated early symptom improvement and resolution in several other immune-related adverse events (irAEs), including ir-colitis, ir-hepatitis, hematological irAEs, ir-arthritis, and ir-myositis [51]. Regarding ICI-related pancreatitis, the evidence remains limited. In a recent collection of case series and clinical studies on Pembrolizumab-induced pancreatitis, the therapeutic options used for steroid-refractory acute pancreatitis (AP) have included Infliximab, Nafamostat mesylate, Azathioprine, and Mycophenolate mofetil [52]. Ohwada S. et al. reported a case in which the administration of Infliximab 5 mg/kg IV showed a reduction in pancreatic enzyme levels and also an improvement in the CT imaging after the third administration [53]. Some case reports suggest that Azathioprine and Mycophenolate Mofetil may be used as maintenance therapy for patients who experience a relapse after steroid withdrawal [54]. This approach is supported by clinical and imaging similarities between ICI-associated pancreatitis and autoimmune pancreatitis, both of which are mediated by T-helper cells. Given that Azathioprine is routinely used in autoimmune pancreatitis to prevent relapse following initial steroid treatment, its potential role in managing ICI-associated pancreatitis is further reinforced [55]. Santoro et al. explored the use of a Rituximab regimen in ICI-AP, given its established role in autoimmune pancreatitis (AIP), and observed remarkable results, with both clinical and radiological improvement occurring just two weeks after the first infusion [55]. Nevertheless, there is a paucity of data that substantiates the application of immunosuppressive agents in ICI-associated pancreatitis, and the possible unfavorable effects of immunosuppressants on ICI efficacy are barely considered [56]. A crucial concern is the potential imbalance between effective immunosuppression and preservation of antitumor immunity. For instance, steroid use for irAEs does not appear to compromise cancer outcomes in many settings [57], although baseline steroid use for comorbidities has been associated with poorer survival [58]. In a study involving 185 patients treated with Infliximab for immune adverse events, it was not associated with a significant increase in tumor progression overall, although a marginal association with reduced survival was noted in the subgroup of patients with genitourinary malignancies [59]. Moreover, a major concern remains the rechallenge of the same ICI in patients who have experienced ICI-induced pancreatitis. However, a recent multicenter retrospective study revealed that the recurrence of ICI-induced pancreatic injury in these patients is a rare event and, when it occurs, it can be successfully managed with ICI discontinuation and steroid therapy [27]. It is also advisable to consult a specialized pancreatic expert before proceeding with therapy. While the NCCN guidelines recommend evaluating ICI rechallenge after the clinical and radiological resolution of pancreatitis, along with improvements in amylase and lipase levels, careful consideration is essential. In cases of severe pancreatitis, rechallenge is not indicated, as recurrent severe pancreatitis can have life-threatening consequences [47].

## 6. Outcomes

ICI-associated pancreatitis can result in the development of chronic pancreatitis, which may lead to secondary pancreatic insufficiency affecting both endocrine and exocrine functions [60]. Indeed, ICI-associated pancreatitis may be characterized by pancreatic volume loss, with over half of patients experiencing >20% atrophy during the first year after initial presentation, particularly in those receiving a combined CTLA-4 and PD-1/PD-L1 blockade [60]. A retrospective analysis of 25 patients revealed that approximately 44% developed pancreatic parenchymal atrophy following ICI-associated pancreatitis, though symptoms of pancreatic insufficiency were observed only in 36% of these patients [40]. Notably, exocrine pancreatic insufficiency can develop as a complication of ICI treatment even in the absence of radiological signs of chronic pancreatitis, although the specific type of ICI, duration of ICI therapy, and total ICI doses administered have not yet been clearly identified as established risk factors [61,62]. A retrospective study of patients with cancer treated with anti-CTLA-4 and/or anti-PD-(L)1 therapies reported an incidence of ICI-induced exocrine pancreatic insufficiency of 1.18 cases per 1000 person-years, occurring without radiological features of chronic pancreatitis [61]. Interestingly, only 39% of patients had a history of acute pancreatitis, suggesting that, while pancreatitis may increase the risk, it is not a prerequisite for the development of EPI, which can also occur independently [61]. Additionally, patients with EPI showed a higher rate of new-onset or worsening diabetes compared with controls [61]. Since clinical signs of EPI, such as steatorrhea, weight loss, and abdominal pain, overlap with those of ir-colitis, EPI may be mistaken for colitis [61]. Notably, the median time to onset of EPI following ICI initiation exceeds 12 months, which is significantly longer than that of ir-colitis, typically occurring around 2 months after starting ICI therapy [61]. Therefore, a case-by-case exclusion of ir-colitis is necessary to ensure accurate diagnosis [62]. There are no specific guidelines for the treatment of EPI following ICIs. In two case reports, patients were treated with steroids, but there was no clinical improvement, while supplementation with pancreatic enzymes resulted in symptom relief [63]. In addition, endocrine insufficiency is also a significant concern. A retrospective study conducted over 10 years at Roswell Park Comprehensive Cancer Center found that the incidence of ICI-induced diabetes mellitus was approximately 1.01% [64]. This complication is typically associated with the use of PD-1 or PD-L1 checkpoint inhibitors rather than anti-CTLA-4 therapy. Furthermore, a recent meta-analysis identified immune checkpoint inhibitor (ICI)-induced diabetes mellitus (DM) as the most commonly reported long-term adverse event, with incidence rates up to 1.27% after ICI therapy. Notably, more than a half of these cases present with diabetic ketoacidosis (DKA), a potentially life-threatening complication that necessitates prompt hospitalization [65]. However, it is important to note that pathogenesis of ICI-induced diabetes involves various underlying mechanisms and the contribution of pancreatitis is only a small part of the overall issue [66]. ICIs can cause pancreatic inflammation, leading to exocrine dysfunction and, in some cases, the development of diabetes, but also directly induce the immune-mediated destruction of pancreatic β cells through the dysregulation of the PD-1/PD-L1 signaling pathway [67]. In addition, genetic predisposition appears to play a role in the development of ICI-induced DM. Specific HLA haplotypes and polymorphisms in the CTLA-4 and PD-1 genes have been associated with an increased risk of autoimmune β cell destruction and the onset of type 1 diabetes mellitus (T1DM) following ICI therapy [66]. Furthermore, among the reported complications of ICI-induced pancreatitis, one case involved a fistulous communication between a pancreatic tail collection and the splenic flexure of the colon [68]. These findings underscore the importance of a balanced and individualized approach when managing ICI-induced pancreatitis. Corticosteroid therapy should be initiated when clinically warranted to control pancreatic inflammation; however, clinicians must be aware of the potential for secondary complications. Careful risk-benefit evaluation and close patient monitoring are crucial to preventing severe or life-threatening outcomes.

## 7. Discussion

**Figure 2:**
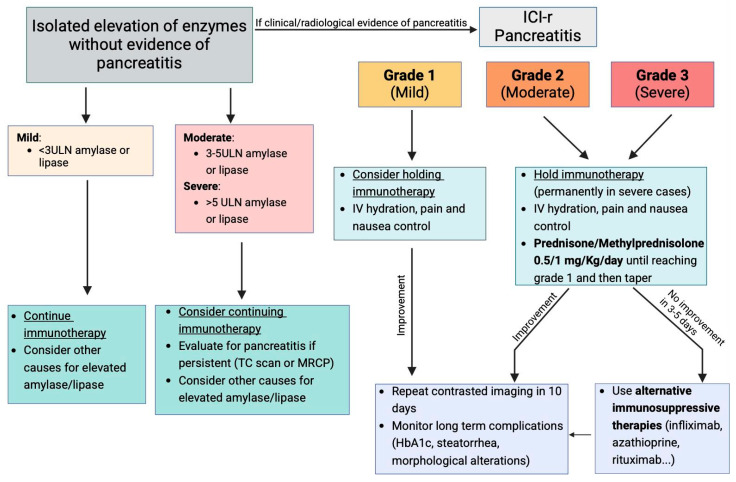
Management algorithm for ICI-related pancreatitis and isolated enzyme elevation.

The increasing use of ICIs in cancer treatment has significantly expanded therapeutic options for patients, particularly in advanced and metastatic cancers [69]. However, as the use of ICIs rises, so does the need for a clear understanding and management of their irAEs, including pancreatic injury [11]. Pancreatic injury due to ICIs, although rare, has been linked to serious complications, including pancreatitis and pancreatic insufficiency, which can severely impact the patient’s quality of life [27]. As a result, the establishment of management guidelines based on large-scale clinical studies rather than anecdotal experience is essential to provide effective care while minimizing unnecessary drug discontinuation [47]. Figure 2 describes the management for ICI-related pancreatitis and isolated enzyme elevation. Routine monitoring in asymptomatic patients is generally not recommended and should only be conducted when there is a suspicion of pancreatitis [35]. An essential distinction in the management of ICI-induced pancreatic injury is the differentiation between asymptomatic elevations of pancreatic enzymes and the development of pancreatitis [70]. Isolated increases in serum amylase and lipase may occur during ICI therapy, but these should not immediately be interpreted as signs of clinically significant pancreatitis. If the increase in enzyme levels is less than three times the upper limit of normal, there is no indication to suspend ICI therapy. In such cases, other causes of enzyme elevation should be explored [47]. However, when enzyme levels increase moderately (between three and five times the upper limit of normal) or severely (more than five times the upper limit), closer evaluation is warranted. In these instances, while ICI therapy may still be continued, it is crucial to perform imaging studies such as a computed tomography (CT) scan or magnetic resonance imaging (MRI) to rule out the presence of pancreatitis or any other pancreatic pathology, which could require a change in treatment approach. In cases where there is an elevated enzyme level along with clinical symptoms (e.g., abdominal pain, nausea, or vomiting) or radiologic findings indicative of pancreatitis, the diagnosis of ICI-AP must be considered [47]. It is critical to distinguish between the severity of pancreatitis to guide management. The classification of pancreatitis can be done in three stages: mild, moderate, and severe.

- **Mild Pancreatitis**: In these cases, ICIs should be temporarily discontinued, and supportive care, including hydration and control of pain and nausea, should be initiated. If the clinical condition improves with supportive measures, a follow-up radiologic exam should be conducted in 10 days. Long-term complications such as diabetes or steatorrhea should also be evaluated. If the patient continues to improve and no signs of severe complications emerge, the ICIs may potentially be resumed [47].
- **Moderate or Severe Pancreatitis**: These conditions warrant the immediate and permanent discontinuation of ICI therapy, particularly in severe pancreatitis, where rechallenge with the same ICI is not recommended [47]. In these cases, patients should receive fluid resuscitation, pain management, and anti-nausea treatment, with glucocorticosteroids initiated at a dose of 0.5–1 mg/kg/day for moderate pancreatitis and 1–2 mg/kg/day for severe pancreatitis, although the response to steroids is not always optimal. If no significant clinical improvement is observed within 3–5 days, additional immunosuppressive treatments may be considered. Thus, alternative immunosuppressive agents, such as TNF-α antagonists, have shown promise in treating steroid-refractory ICI-AP [53]. Infliximab, for instance, has been used successfully in cases of ICI-associated colitis, hepatitis, and myositis, and preliminary evidence suggests it may improve outcomes in ICI-AP as well. In cases of relapse following steroid withdrawal, agents like azathioprine and mycophenolate mofetil, which are commonly used in autoimmune pancreatitis, may be considered as maintenance therapies [54]. This approach is supported by the similarities between ICI-associated pancreatitis and autoimmune pancreatitis, both of which are believed to be T-helper cell-mediated processes.

## 8. Conclusions

In conclusion, considering the expected rise in the use of immune ICIs, it is crucial to increase awareness about the potential for pancreatic injury associated with these treatments. The differentiation between isolated elevations in amylase and lipase and clinically significant pancreatitis is key to ensuring appropriate intervention. This will help to ensure that management protocols are clearly defined based on large-scale and evidence-based studies. Such efforts will contribute to more effective and standardized care for patients undergoing ICI therapy, with the goal of appropriately managing these complications and avoiding unnecessary or improper discontinuation of the medication.

## Figures and Tables

**Table 1:** Incidence of pancreatic injury and pancreatitis with ICIs.

Author, Year, Study Type	Therapy Type	Inhibitor Class	All-Grade PI (%)	Grade ≥ 3 PI (%)	Notes
George et al. (2019) [16] Systematic review and meta-analysis	Monotherapy	PD-1	0.94% (95% CI: 0.48–1.40)	Not specified	Lower PI incidence compared with CTLA-4 inhibitors
Su et al. (2018) [20] Systematic review and meta-analysis	Monotherapy	CTLA-4	3.98% (95% CI: 2.92–5.05)	Not specified	Higher PI incidence than PD-1 inhibitors
Hofmann et al. (2016) [22] Retrospective study	Combination	PD-1 + CTLA-4	10.6% (95% CI: 7.89–13.32)	Not specified	Additive increase in PI incidence with combination therapy
Zhou et al. (2021) [21] Systematic review and meta-analysis	Monotherapy	PD-1	2.0% (95% CI: 1.67–2.39)	1.8% (95% CI: 1.41–2.29)	Lower severe PI incidence
Bagchi et al. (2021) [23] Review	Monotherapy	PD-L1	3.01% (95% CI: 1.86–4.87)	3.1% (95% CI: 1.7–5.64)	Higher PI incidence compared with PD-1 inhibitors
Thompson et al. (2020) [26] Guidelines	Monotherapy	CTLA-4	2.92% (95% CI: 0.99–8.65)	2.69% (95% CI: 0.76–9.49)	PI incidence similar to PD-L1 inhibitors

**Table 2:** Comparison of ICI-related pancreatitis and isolated enzyme elevation.

Feature	True irAE Pancreatitis	Benign Enzyme Elevation
**Lipase/Amylase Levels**	>3× ULN (often >5× ULN)	Mild/moderate elevation, often <3× ULN
**Abdominal Symptoms**	Present (e.g., epigastric pain, nausea, vomiting)	Absent
**Imaging (CT/MRI/US)**	Findings consistent with pancreatitis (edema, swelling)	Normal
**Timing**	Typically, within 4–12 weeks of ICI initiation	Variable; may be incidental
**Other Laboratory Abnormalities**	↑ CRP, ↑ WBC, possible ↑ transaminases or bilirubin	None or mild inflammatory markers
**Clinical Course**	May require hospitalization and immunosuppression	Self-limiting; often resolves spontaneously
**Management**	Steroids ± ICI interruption; imaging follow-up needed	Monitoring only; no need for steroids or imaging

## Data Availability

Not applicable.

## Abbreviations

(ICIs)	Immune checkpoint inhibitors
(ICI-PI)	ICI-induced pancreatic injury
(irAEs)	Immune-related adverse events
(EPI)	Exocrine pancreatic insufficiency
(AIP)	Autoimmune pancreatitis
(ICI-AP)	ICI-associated pancreatitis
(CTLA-4)	CytotoxicT-lymphocyte antigen 4
(PD-1)	Programmed cell death 1
(PD-L1)	Ligand of programmed cell death 1
